# Prerequisites for amplicon pyrosequencing of microbial methanol utilizers in the environment

**DOI:** 10.3389/fmicb.2013.00268

**Published:** 2013-09-05

**Authors:** Steffen Kolb, Astrid Stacheter

**Affiliations:** Department of Ecological Microbiology, University of BayreuthBayreuth, Germany

**Keywords:** methylotroph, PQQ MDH, PQQ MDH2, NAD MDH, FAD AO, *mtaC*, *xoxF*, *mxaF*^′^

## Abstract

The commercial availability of next generation sequencing (NGS) technologies facilitated the assessment of functional groups of microorganisms in the environment with high coverage, resolution, and reproducibility. Soil methylotrophs were among the first microorganisms in the environment that were assessed with molecular tools, and nowadays, as well with NGS technologies. Studies in the past years re-attracted notice to the pivotal role of methylotrophs in global conversions of methanol, which mainly originates from plants, and is involved in oxidative reactions and ozone formation in the atmosphere. Aerobic methanol utilizers belong to Bacteria, yeasts, Ascomycota, and molds. Numerous bacterial methylotrophs are facultatively aerobic, and also contribute to anaerobic methanol oxidation in the environment, whereas strict anaerobic methanol utilizers belong to methanogens and acetogens. The diversity of enzymes catalyzing the initial oxidation of methanol is considerable, and comprises at least five different enzyme types in aerobes, and one in strict anaerobes. Only the gene of the large subunit of pyrroloquinoline quinone (PQQ)-dependent methanol dehydrogenase (MDH; *mxaF*) has been analyzed by environmental pyrosequencing. To enable a comprehensive assessment of methanol utilizers in the environment, new primers targeting genes of the PQQ MDH in *Methylibium* (*mdh2*), of the nicotinamide adenine dinucleotide-dependent MDH (*mdh*), of the methanol oxidoreductase of Actinobacteria (mdo), of the fungal flavin adenine nucleotide-dependent alcohol oxidase (*mod1*, *mod2*, and homologs), and of the gene of the large subunit of the methanol:corrinoid methyltransferases (*mtaC*) in methanogens and acetogens need to be developed. Combined stable isotope probing of nucleic acids or proteins with amplicon-based NGS are straightforward approaches to reveal insights into functions of certain methylotrophic taxa in the global methanol cycle.

## PREFACE

The commercial launch of pyrosequencing and later on, further next generation sequencing (NGS) technologies for direct sequencing of complex PCR amplicons facilitated the assessment of microbial communities by geno- and ribotype composition with high coverage and resolution, and with a high number of samples (e.g., Pilloni et al., 2012). Methylotrophic bacteria were among the first group of microorganisms that were analyzed by molecular tools in the environment (Holmes et al., 1995). Based on such studies substantially more is known on the pivotal role of microorganisms for global cycles of one-carbon (C1) compounds, such as the greenhouse gas methane. Another quantitatively important atmospheric volatile organic compound (VOC) is methanol. Nonetheless, *in situ* activities, environmental drivers, and distribution of methanol utilizers in the environment has scarcely been addressed (e.g., McDonald and Murrell, 1997), and just few years ago, studies re-attracted notice to activities of methanol utilizers in the environment (Delmotte et al., 2009; Dixon et al., 2011).

## METHYLOTROPHS IMPACT ON GLOBAL ONE-CARBON COMPOUND CYCLING

Aerobic methylotrophs occur in terrestrial and aquatic environments on the whole planet, and have been detected in aerated and flooded soils of wetlands, grasslands, tundra, and deserts, and occur in the phyllosphere and rhizosphere of plants, in open ocean waters and other marine habitats (Giovannoni et al., 2008; Angel and Conrad, 2009; Kolb, 2009a; Wieczorek et al., 2011; Bissett et al., 2012; Gupta et al., 2012; He et al., 2012; Knief et al., 2012a; Vorholt, 2012) suggesting that their unique physiology that allows them to utilize reduced C1 compounds as carbon and energy source is of global relevance in ecosystems. Methylotrophs ubiquitously occur in terrestrial ecosystems, i.e., likely, since plants produce C1 compounds. Growing plants emit methanol (up to 0.1% of the photosynthetic carbon) and traces of chloromethane and methane (Keppler et al., 2005; Keppler et al., 2006), during decay of lignocellulosic plant material methanol is released (Galbally and Kirstine, 2002), and plant compounds are eventually converted to methane under anoxic conditions (Drake et al., 2009). C1 compounds are highly volatile and thus, are emitted into the atmosphere. Consequently, the two most abundant organic compounds in the atmosphere are methane and methanol (Forster et al., 2007). The steady-state concentration of methanol in the atmosphere (1–10 ppb) is about 1,000-fold lower than that of atmospheric methane (1,800 ppb; Galbally and Kirstine, 2002; Heikes et al., 2002; Jacob et al., 2005). Whereas, the estimated global emission rate of methane (~10 Tmol per year) from terrestrial ecosystems is only twice as high as the global terrestrial emission rate of methanol (~5 Tmol per year) indicating that methanol is substantially more susceptible to atmospheric chemical reactions (Galbally and Kirstine, 2002; Jacob et al., 2005; Kolb, 2009a). Methanol triggers the formation of tropospheric ozone, and has indirectly a threefold higher global warming potential on a one-hundred-year basis than carbon dioxide (Forster et al., 2007).

Release of methane from terrestrial ecosystems into the atmosphere is reduced by aerobic methylotrophs (Conrad, 1995). Many aerated soils in natural ecosystems are even net sinks for atmospheric methane, which is often correlated with the predominance of certain genotypes, such as USCα (Dunfield, 2007; Kolb, 2009b). Methanotrophic methylotrophs have been addressed in numerous environmental studies by using gene markers and other biomarkers, and are one of the most studied functional groups of microorganisms in the environment (e.g., Dedysh, 2009; Kolb, 2009b; Dörr et al., 2010; Lüke et al., 2010; Lüke and Frenzel, 2011). There are more than 400 publications on methanotrophs in ecosystems over the past 25 years based on keyword searches in literature databases (Web of Knowledge, 04.07.2013, ) highlighting the interest in understanding the role of methanotrophs in the global carbon cycle.

Non-methanotrophic methylotrophs likely have a similar importance for the global methanol cycle, a fact that has recently been more thoroughly addressed in the phyllosphere, soil, and ocean waters (Delmotte et al., 2009; Kolb, 2009a; Knief et al., 2010a, b; Dixon et al., 2011, 2013; Vorholt, 2012; Stacheter et al., 2013). The assessment of methanol-utilizing methylotrophs in the environment is less straightforward than the detection of methanotrophs, since methanol utilizers have a substantially larger diversity than methanotrophs, and the enzymes that catalyze the diagnostic reaction, i.e., the oxidation of methanol to formaldehyde, are more diverse than methane monooxygenases making the detection of non-methanotrophic methanol utilizers more challenging (Chistoserdova et al., 2009; Kolb, 2009a; Stacheter et al., 2013).

The role of methanol utilizers in global methanol cycling is still scarcely investigated and warrants studies that address the response, activity, and distribution of methanol utilizers in terrestrial and other environments. Hence, suitable gene targets are mandatory to analyze methanol-utilizing microorganisms with amplicon pyrosequencing or to detect them in metagenomic, transcriptomic, and proteomic datasets based on sequence homology. The review will describe the latest knowledge on microbial taxa that are capable of methanol oxidation including those organisms that putatively utilize methanol under anoxic conditions, and will identify gene markers that have been and can be employed for analysis of PCR amplicons by high-throughput NGS techniques.

## FACULTATIVELY AEROBIC METHANOL UTILIZERS

Microorganisms that have the capability to utilize methanol with molecular oxygen as an electron acceptor belong to various phyla of Bacteria, and have been found within yeast, mold fungi, and Ascomycota (**Table 1**; Bystrykh et al., 1988; Trotsenko and Bystrykh, 1990; Nakagawa et al., 1996; Nozaki et al., 1996; Silva et al., 2009; Sipiczki, 2012). Bacterial methanol utilizers belong to Alphaproteobacteria, Gammaproteobacteria, Betaproteobacteria, Flavobacteriia, Bacilli, and Actinobacteria. Yet, methanol utilization (MUT) among Archaea only occurs in strict anaerobic methanogens. Generally, it is known that several bacterial methylotrophs utilize methanol or other C1 compounds for dissimilation, but cannot assimilate carbon from C1 compounds. Strain HTCC2181 is a recent example, which demonstrates this strategy of C1 compound utilization (Giovannoni et al., 2008; Halsey et al., 2012). Over 200 aerobic species of methylotrophic Bacteria have been described (**Tables 1** and **2**; Kolb, 2009a). Most of the known isolates are Gram-negative. Thus, it is remarkable that a second isolate of the genus *Bacillus* has recently been described, which was not enriched on conventional methylotroph media suggesting a largely uncovered diversity of Gram-positive methanol utilizers in the environment (**Table 2**; Ling et al., 2011).

**Table 1:** Classes and phyla of Bacteria and fungi that contain methanol-utilizing methylotrophs based on previous reviews (Kolb, 2009a; Gvozdev etal., 2012).

Class/phylum/order	Representative species
**Actinobacteria**
Brevibacteriaceae	*Brevibacterium casei*
Micrococcaceae	*Arthrobacter methylotrophus*
Mycobacteriaceae	*Mycobacterium gastri*
Nocardiaceae	*Rhodococcus erythropolis*
Pseudonocardiaceae	*Amycolatopsis methanolica*
**Bacilli**
Bacillaceae	*Bacillus methanolicus*
**Alphaproteobacteria**
Acetobacteraceae	*Acidomonas methanolica*
Beijerinckiaceae	*Methylocapsa aurea*
Bradyrhizobiaceae	*Afipia felis*
Hyphomicrobiaceae	*Angulomicrobium tetraedrale*
Methylobacteriaceae	*Methylobacterium extorquens*
Methylocystaceae	*Methlyopila jiangsuensis*
Phyllobacteriaceae	*Mesorhizobium loti*^A^
Rhizobiaceae	*Ensifer fredii*^A^
Rhodobacteraceae	*Paracoccus alkenifer*
Sphingomonadaceae	*Sphingomonas melonis*
Xanthobacteraceae	*Ancylobacter dichloromethanicus*
**Betaproteobacteria**
Comomonadaceae	*Variovorax paradoxus*
Methylophilaceae	*Methylophilus glucosoxydans*
Rhodocyclaceae	*Methyloversatilis universalis*
Burkholderiales	*Methylibium aquaticum*
**Gammaproteobacteria**
Enterobacteriaceae	*Klebsiella oxytoca*
Methylococcaceae	*Methylococcus capsulatus*
Piscirickettsiaceae	*Methylophaga thiooxydans*
Vibrionaceae	*Photobacterium indicum*
Classification unclear	*Methylohalomonas lacus*
**Ascomycota**
	*Gliocladium deliquescens*
	*Paecilomyces variotii*
	*Trichoderma lignorum*
**Yeasts**
	*Candida boidini *(and others)
	*Hansenula capsulatus *(and others)
	*Pichia pastoris *(and others)
**Mold fungi**
	*Paecilomyces variotii*
	*Penicillium chrysogenum*

A Harbors *xoxF*-like gene *mxaF*^′^. Growth on methanol has not been tested.

**Table 2:** List of methanol-utilizing methylotophs that are not included in a previous survey (Kolb, 2009a).

Class/phylum	Isolation source	fac	pH	Reference
**Actinobacteria**
*Micrococcus luteus* MM7	Oral Cavity	+	nn	Anesti et al. (2005)
**Bacilli**
*Bacillus vallismortis* JY3A	Soil	+	N	Ling et al. (2011)
**Alphaproteobacteria**
*Ancylobacter dichloromethanicus* DM16T	Soil	+	N	Firsova et al. (2009)
*Ancylobacter oerskovii* NS05	Soil	+	N	Lang et al. (2008)
*Ancylobacter polymorphus* DSM 2457	Soil	+	N	Xin et al. (2006)
*Ancylobacter rudongensis* JCM 1167	Rhizosphere	+	N	Xin et al. (2004)
*Ancylobacter vacuolatus* DSM 1277	Soil	+	N	Xin et al. (2006)
*Methylobacterium bullatum* B3.2	Phyllosphere	+	N	Hoppe et al. (2011)
*Methylobacterium cerastii* C15	Phyllosphere	+	Act	Wellner et al. (2012)
*Methylobacterium gnaphalii* AB627071	Phyllosphere	+	nn	Tani et al. (2012a)
*Methylobacterium goesingense* AY364020	Rhizosphere	+	N	Idris et al. (2006)
*Methylobacterium gossipiicola* Gh-105	Phyllosphere	+	N	Madhaiyan et al. (2012)
*Methylobacterium longum* DSM 23933	Phyllosphere	+	N	Knief etal. (2012a, b)
*Methylobacterium marchantiae* DSM 21328	Phyllosphere	+	N	Schauer et al. (2011)
*Methylobacterium oxalidis* DSM 24028	Phyllosphere	+	N	Tani et al. (2012b)
*Methylobacterium phyllosphaera* BMB27	Phyllosphere	+	N	Madhaiyan et al. (2009a)
*Methylocapsa aurea* DSM 22158	Soil	+	Act	Dunfield et al. (2010)
*Methyloferula stellata* AR4	Soil	-	Ac	Vorobev et al. (2011)
*Methlyopila jiangsuensis* DSM 22718	Activated sludge	+	N	Li et al. (2011)
*Starkeya koreensis* Jip08	Phyllosphere	+	N	Im et al. (2006)
*Starkeya novella* IAM 12100	Phyllosphere	+	N	Im et al. (2006)
**Betaproteobacteria**
*Methylobacillus arboreus* VKM B-2590	Phyllosphere	+	N	Gogleva et al. (2011)
*Methylobacillus gramineus* VKM B-2591	Phyllosphere	+	N	Gogleva et al. (2011)
*Methylopila musalis* MUSAT	Banana	+	N	Doronina et al. (2013)
*Methylophilus rhizosphaerae* BMB147	Rhizosphere	+	N	Madhaiyan et al. (2009b)
*Methylophilus glucosoxydans* B	Rhizosphere	+	N	Doronina et al. (2012)
*Methylophilus flavus* DSM 23073	Phyllosphere	-	N	Gogleva et al. (2010)
*Methylophilus luteus* DSM 2949	Phyllosphere	+	N	Gogleva et al. (2010)
*Methylotenera versatilis* JCM 17579	Sediment	+	N	Kalyuzhnaya et al. (2012)
*Methylovorus menthalis* DSM 24715	Rhizosphere	-	Al	Doronina et al. (2011)
*Variovorax paradoxus* 5KTg	Oral Cavity	+	nn	Anesti et al. (2005)
**Gammaproteobacteria**
*Methylomonas koyamae* MG30	Water of rice paddy	-	Act	Ogiso et al. (2012)
*Methylomonas scandinavica* SR5	Groundwater	-	N	Kalyuzhnaya et al. (1999)
*Methylomonas paludis* MG30	Torfmoor	-	Act	Danilova et al. (2013)
*Methylothermus subterraneus* DSM 19750	Aquifer	-	Act	Hirayama et al. (2011)
*Methylophaga lonarensis* MPL	Sediment	-	Al	Antony et al. (2012b)
*Methylophaga sulfidovorans* RB-1	Sediment	-	N	de Zwart et al. (1996)
*Methylophaga thiooxydans* DSMO10	Marine waters	+	nn	Boden et al. (2010)

*fac, facultative methylotrophic; +, growth in substrates with carbon –carbon bonds; -, no growth in substrates with carbon –carbon bonds; nn, no information in species description; enrichment at pH 7; N, neutrophile (growth optimum between pH 6.5 and 8.0); Act, acid tolerant (growth optimum between pH 3.0 und 6.5); Ac, acidiphilic (growth optimum below pH 3); Al, alcaliphilic (growth optimum over pH 8).*

**FIGURE 1:**
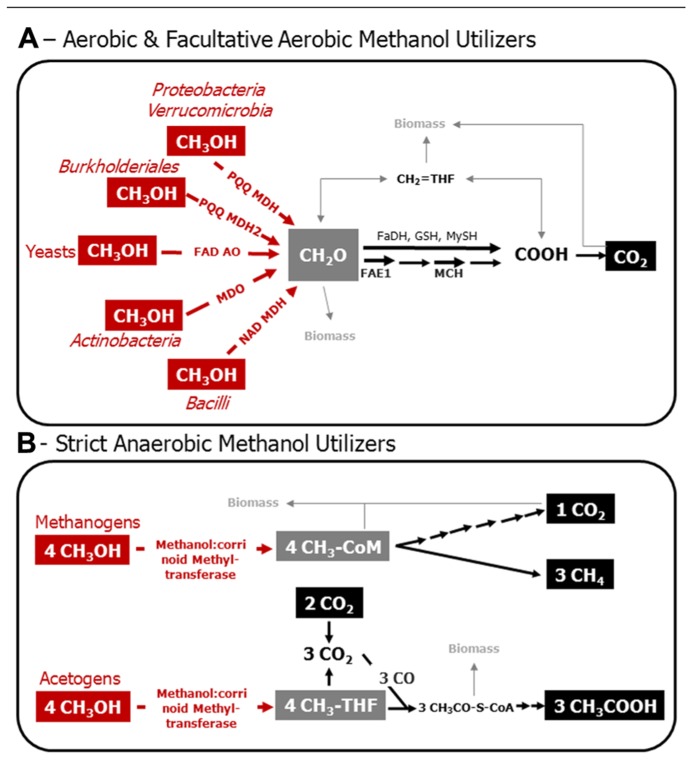
**Model of the C1 metabolism of aerobic and facultatively aerobic methanol utilizers (A), and of methanol-utilizing methanogens and acetogens (B).** Various Bacteria that employ PQQ MDH and PQQ MDH2 utilize nitrate besides molecular oxygen, and are, thus, facultative aerobes. Red, enzymatic reactions indicative for methanol utilization. Gray, metabolic crossing points to anabolic pathways. Structural genes are targets for the molecular assessment of methanol-utilizing microorganisms in the environment. PQQ MDH, PQQ-dependent methanol dehydrogenase; PQQ MDH2, alternative PQQ-dependent methanol dehydrogenase in *Methylibium* andBurkholderia strains; FAD AO, FAD-dependent alcohol oxidoreductase; MDO, methanol oxidoreductase; NAD MDH, NAD-dependent methanol dehydrogenase; FAE1, tetrahydromethanopterin-dependent formaldehyde activating enzyme; MCH, tetrahydromethanopterin-dependent methenyl-methylene cyclohydrolase; FaDH, formaldehyde dehydrogenase; GSH, glutathione-dependent formaldehyde oxidation; MySH, mycothiol-dependent oxidation of formaldehyde in yeast; CH_2_ = HF4, methylene tetrahydrofolate; CH_3_-THF, methyl-tetrahydrofolate; CH_3_-CoM, methyl-coenzyme M; CH_3_OH, methanol; CH_2_O, formaldehyde; COOH formate; CO_2_, carbon dioxide; CO, carbon monoxide; CH_3_CO–S–CoA, acetyl coenzyme A; CH_3_COOH, acetic acid. Biomass, assimilation of carbon occurs at the level of formaldehyde and/or on carbon dioxide in aerobic methylotrophs; pathways involved are the Calvin–Benson–Bassham cycle, ribulose-monophosphate pathway, and the serine cycle. Assimilation of carbon in methanogens is mediated by a unique reductive acetyl-CoA-pathway, whereas acetogens form acetyl-CoA as an intermediate that can be used for biosnthesis. This figure is based on previous articles (Thauer, 1998; Ding et al., 2002; Hagemeier et al., 2006; Das et al., 2007; Drake et al., 2008; Chistoserdova et al., 2009; Chistoserdova, 2011; Gvozdev et al., 2012).

It is well established that some facultatively aerobic methanol utilizers are capable of growth on C1 compounds with nitrate as an electron acceptor (Kolb, 2009a). In addition, many more methylotrophs that have the ability to use nitrate as an alternative electron acceptor have not yet been tested for anaerobic methanol oxidation (Bamforth and Quayle, 1978; Kolb, 2009a); recent examples, in which the physiology has been thoroughly assessed, are *Methyloversatilis universalis* FAM5, and *Methylotenera versatilis* (Kalyuzhnaya et al., 2012; Lu et al., 2012; Mustakhimov et al., 2013). In environments with a high nitrogen input (for example by fertilization) and turnover, facultative aerobic and nitrate-dependent degradation of methanol likely occurs in oxygen-limited zones (Lu et al., 2012). Based on the current knowledge, these organisms are accessible by the same gene markers as described in the following section (**Figure 1**).

## MARKER GENES OF BACTERIAL METHANOL UTILIZERS

Amplicon-based analysis of the diversity of methanol utilizers can be achieved by deep sequencing of genes that are diagnostic for methanol oxidation (**Figure 1**; Stacheter et al., 2013). The C1 metabolism of bacterial methanol utilizers comprises a series of enzymatic reactions, which partially cannot be found in other heterotrophs and are thus, diagnostic for methylotrophs. The most characteristic enzymatic step is the initial oxidation of methanol to formaldehyde (**Figure 1**). The oxidation of methanol can be catalyzed by at least three different enzymes in Bacteria. There is a pyrroloquinoline quinone (PQQ)-dependent and a nicotinamide adenine dinucleotide (NAD)-dependent methanol dehydrogenase (MDH; Devries et al., 1992; Chistoserdova et al., 2009; Krog et al., 2013). PQQ MDH occurs in Gram-negative Bacteria, whereas the NAD MDH is typical of Gram-positive *Bacillus* strains and is encoded by the gene *mdh* (Chistoserdova et al., 2009). Furthermore, in Gram-positive Actinobacteria (*Amycolatopsis* *methanolica*, *Mycobacterium gastri* MB19) a methanol:NDMA (*N*, *N*′-dimethyl-4-nitrosoaniline) oxidoreductase (MDO) has been reported (Bystrykh et al., 1993; Vanophem et al., 1993; Park et al., 2010).

The gene *mxaF* encodes the catalytic subunit of PQQ MDH, which is composed of two different subunits (i.e., MxaFI). However, there is a distantly related homolog in some methylotrophic Burkholderiales (*Methylibium*; i.e., the gene was named *mdh2*), which encodes an alternative PQQ-dependent MDH (Kalyuzhnaya et al., 2008). Beyond *mdh2*, a further homolog of *mxaF* is known, i.e., *xoxF*, for which a functional role in methanol-metabolism is under debate. *xoxF*-like genes (synonymous to *mxaF*′) occur in Bradyrhizobiaceae and other rhizobia, and may encode functional MDHs (Fitriyanto et al., 2011). Similar genes can be frequently detected in soil microbial communities using *mxaF*-specific primers (**Table 1**; Stacheter et al., 2013). If rhizobia that do not possess the classical PQQ MDH (i.e., MxaFI; Moosvi et al., 2005), also utilize and grow on methanol has not systematically been analyzed yet. Nonetheless, *Bradyrhizobium* sp. MAFF211645 contains a Ce^3^^+^-inducible XoxF-like MDH (Fitriyanto et al., 2011). The first functional proof of XoxF as a MDH was demonstrated in the phototroph *Rhodobacter sphaeroides* (Wilson et al., 2008). Studies on *Methylobacterium extorquens* AM1 suggest that XoxF1 and XoxF2 are involved in the regulation of *mxaF* (Schmidt et al., 2010; Skovran et al., 2011). Purified XoxF1 of *Methylobacterium extorquens* AM1 has highest methanol oxidation activities when cells were grown with methanol and 30 μM La^3^^+^. These activities were comparable to the canonical PQQ MDH MxaFI. The purified XoxF enzyme contained La^3^^+^ suggesting that XoxF is important as a calcium-independent MDH that uses rare earth elements as cofactors (Nakagawa et al., 2012). In recently discovered methanotrophs of the phylum Verrucomicrobia, *xoxF* is the only detectable gene that may code for MDH (Op den Camp et al., 2009). *xoxF* also occurs in non-methylotrophic bacteria, in which its metabolic function is unresolved (Chistoserdova, 2011). Thus, the detection of *xoxF* by NGS in environmental gene surveys or their occurrence in metagenomes, transcriptomes, and proteomes may be a hint to environmental methanol oxidation but need to be carefully evaluated based on recent and upcoming results from pure cultures of various organisms.

A comprehensive assessment of the genotypic diversity of aerobic methanol utilizers in the environment seems possible when *mxaF*, *xoxF*-like, *mdh2*, *mdh*, and genes of MDO of Actinobacteria are simultaneously analyzed. However, only *mxaF* has been successfully detected to date and PCR primers suitable for environmental surveys of the other genes have not yet been developed (McDonald and Murrell, 1997; Neufeld et al., 2007; Stacheter et al., 2013). More studies on the function of *xoxF* in further methylotrophs and addressing the physiological role of *xoxF* in organisms that are currently not known as methylotrophs are warranted to improve the ability to interprete methylotrophy gene-targeting surveys in the environment. The employment of genes of MDHs of Gram-positive methylotrophs will enhance the environmental detectability of methanol utilizers and will aid to understand the role of these largely overlooked methylotrophs for methanol conversion in ecosystems.

In addition to gene markers that are diagnostic for methanol oxidation, the genes *mch* (methenyl:methylene tetrahydromethanopterin cyclohydrolase) and *fae*1 (formaldehyde-activating enzyme) that are indicative for dissimilatory oxidation of formaldehyde by tetrahydromethanopterin-dependent reactions have been used to detect methylotrophs in the environment (Kalyuzhnaya et al., 2004; Kalyuzhnaya and Chistoserdova, 2005; Stacheter et al., 2013; **Table 3**). However, their detection alone does not allow for the conclusion that the detected organisms are methanol utilizers since *fae1* and *mch* also occur in non-methylotrophic heterotrophs (Chistoserdova, 2011; Stacheter et al., 2013).

**Table 3:** Gene markers of methanol-utilizing microorganisms for amplicon-based pyrosequencing or as targets for homology screens in metagenome, -trancriptome, or -proteome datasets.

Methanol utilizers	Enzyme	Function	Gene marker	Primers	Reference	Pyroseq^D^
Proteobacteria	PQQ MDH	MeOH ox.	*mxaF*	1003f/1555r^E^	McDonald and Murrell (1997); Neufeld et al. (2007)	Yes
Proteobacteria, Verrucomicrobia	PQQ MDH	Putatively MeOH ox.	*xoxF*	1003f/1555r^E^	McDonald and Murrell (1997); Neufeld et al. (2007)	Yes
Burkholderiales	PQQ MDH2	MeOH ox.	*mdh2*	Not available^A^	Kalyuzhnaya et al. (2008)	No
Various Proteobacteria, non-methylotrophs	FAE	Other^B^	*fae1*	fae1f/fae1r	Kalyuzhnaya et al. (2004)	Yes
	MCH	Other^B^	*mch*	mch-2a/mch-3	Vorholt et al. (1999); Kalyuzhnaya and Chistoserdova (2005)	Yes
Actinobacteria	MDO	MeOH ox.	*mdo*	Not available^A^	Park et al. (2010)	No
Bacilli	NAD MDH	MeOH ox.	*mdh*	Not available^A^	Devries et al. (1992); Krog et al. (2013)	No
Methylotrophic methanogens	Methanol:CoM methyl-transferase system	MeOH ox.	*mtaC*^C^	Not available^A^	Hagemeier et al. (2006)	No
Methylotrophic acetogens	Methanol:CoM methyl-transferase system-like	MeOH ox.	*mtaC*-like^C^	Not available^A^	Das et al. (2007)	No
Fungi	FAD AO	MeOH ox.	*mod1*, *mod2*, others	Not available^A^	Reid and Fewson (1994); Ozimek et al. (2005), Hartner and Glieder (2006); Nakagawa et al. (2006)	No

*MeOH, methanol oxidation.*

A Primers for this group of genes have not designed and tested in environmental surveys.

B These enzymes do not oxidize methanol, but are involved in formaldehyde oxidation. These enyzmes also occur in methylotrophs that do not use methanol and in non-methylotrophs (Chistoserdova, 2011).

C Homologs of unknown function are present in methanogens (Ding et al., 2002).

D Has been used in amplicon pyrosequencing.

E Detect only *mxaF* and *xoxF*-like genes of *Proteobacteria*.

## ROUTES TOWARD ENVIRONMENTAL DETECTION OF METHYLOTROPHIC YEASTS, MOLDS, AND ASCOMYCOTA

Fungi employ a unique pathway for methanol oxidation, in which methanol is oxidized via formaldehyde and formate to carbon dioxide, i.e., the MUT pathway (Hartner and Glieder, 2006; **Figure 1**). The initial oxidation of methanol is mediated by a flavin adenine nucleotide (FAD) dependent alcohol oxidase (FAD AO) that produces formaldehyde and hydrogen peroxide (Hartner and Glieder, 2006). FAD AO occurs in various genera of yeasts, such as *Candida* and *Pichia*, in molds, and Ascomycota (**Table 1**; Vandenbosch et al., 1992; Gvozdev et al., 2012). Known genes encoding for FAD AOs are *mod1* and *mod2*, however homologs exist of which the function is unresolved (Nakagawa et al., 2006; Gvozdev et al., 2012). The use of genes of FAD AO for the environmental detection of methylotrophic fungi will be still challenging since numerous isoenzymes with likely different kinetic properties exist (**Table 1**; Ito et al., 2007). The role of the diversity and activity of fungal microorganisms for environmental conversion of methanol has scarcely been studied (**Table 1**), and warrants, especially in terrestrial environments, future research.

## POTENTIAL GENE MARKERS OF STRICT ANAEROBIC METHANOL UTILIZERS

The quantitative contribution of anaerobic methanol conversion in soils has scarcely been analyzed (Conrad and Claus, 2005). Beyond facultative aerobic methylotrophs, some strict anaerobes utilize methanol, i.e., methylotrophic methanogens and acetogens. A methanol-utilizing acetogen is *Moorella thermoacetica*, and examples for methanol-utilizing methanogens are *Methanosarcina acetivorans*, *Methanolobus* sp., and *Methanosarcina barkeri* (Das et al., 2007; Antony et al., 2012a; Penger et al., 2012). Recently, the methanol-oxidizing enzyme methanol:corrinoid methyltransferase (MtaC), encoded by *mtaC*, has been structurally characterized in these organisms (Ding et al., 2002; Hagemeier et al., 2006). An enzyme with homology to MtaC is upregulated during growth on methanol in methylotrophic acetogens (Zhou et al., 2005; Das et al., 2007). Hence, the gene *mtaC* and its homolog in acetogens are promising targets to develop gene-based detection of strict anaerobic methanol utilizers in the environment.

## ASSESSMENT OF METHANOL UTILIZERS BY AMPLICON PYROSEQUENCING

The advent of NGS technologies allow for a dramatic increase of sequence information compared with similar efforts when using classic Sanger sequencing (Christen, 2008; Liu et al., 2012). Amplicon pyrosequencing (i.e., a synonymous term is pyrotag sequencing) is one of the best evaluated and oldest NGS technologies (Liu et al., 2012). Long reads of about 700–1000 bp are possible, and technically unavoidable sequence errors can be removed with established software, such as AmpliconNoise (Quince et al., 2011; Rosen et al., 2012). Thus, large datasets with over 100,000 reads can be quality filtered, trimmed, sorted, and clustered into sequence similarity-defined operational taxonomic units (Caporaso et al., 2010; Nebel et al., 2011). Amplicon pyrosequencing comes along with higher costs per read compared to cheaper technologies, such as HiSeq, MiSeq, or Ion Torrent (Liu et al., 2012). However, the long-read length of pyrosequencing is especially advantageous when analyzing amplicons. Beyond that, amplicon-based pyrosequencing generates highly reproducible and similar community structures when compared to standard community fingerprinting techniques, such as terminal restriction fragment length polymorphism (TRFLP) analysis and thus, can be used for reliable genotype composition analyses (Pilloni et al., 2012).

Amplicons can be obtained with primers that contain adapter sequences that are needed for emulsion PCR-based amplification to generate nanobead-bound sequence libraries (Ronaghi et al., 1998). Usually such primers include a several nucleotide-long sequence for the identification of the source of sequence (i.e., a barcode), such as an individual sample within the study (Pilloni et al., 2012). A consequence is long primers with unspecific extensions at 5′ end. This may lead to reduced sensitivity of amplification and to unspecific binding (Berry et al., 2011). One strategy to overcome this bias is applying two subsequent PCRs. The first PCR is conducted with untagged primers followed by a second PCR with tagged primers (Berry et al., 2011). An alternative strategy to minimize these shortcomings is to use primers with barcodes, but without adapter sequences. Adapters need to be added by ligation after the PCR (Stacheter et al., 2013). A complication of such an approach is the retrieval of two datasets one with sequences starting with forward and one starting with the reverse primer, but minimizes bias during amplicon amplification (Stacheter et al., 2013). Environmental detection of *mxaF*-like genotypes of methylotrophs by primers 1003f and 1555r include the detection of *xoxF*-like genes (**Table 3**; Stacheter et al., 2013). When *mxaF*-targeting primers were used, also *xoxF*-like genes were detected in various grassland and forest soils by amplicon pyrosequencing (Stacheter et al., 2013) making the interpretation of data in regard to the capability of detected microorganisms of methanol oxidation more difficult, since the function of *xoxF* is still in part under debate.

## *mxa*F AND HOMOLOGS FOR ENVIRONMENTAL DETECTION OF METHYLOTROPHS

Analysis of non-methanotrophic methylotrophs by *mxaF* genotyping has been employed in several studies. Nonetheless, only one study exist that employed amplicon pyrosequencing (**Table 4**). All other amplicon-based NGS studies addressed methanotrophs and mostly analyzed *pmoA* (i.e., encodes a gene of a subunit of the particulate methane monooxygenase). The use of amplicon pyrosequencing is a great step forward toward complete coverage of the real diversity that exists in a given habitat. In this review, the authors argue in favor to target structural genes of methanol utilizers. One advantage of the use of structural genes is the increased sensitivity since rare groups, such as methylotrophs in soil communities, can be more reliably detected than by a 16S rRNA gene-based survey. Several methylotrophs occur in taxa of which only some members are capable of methylotrophy (e.g., Bacillaceae; **Tables 1** and **2**). The detection of such methylotrophs by 16S rRNA genes can be misleading, and thus, another advantage of the use of genes encoding a methanol-oxidizing enzyme is that the detection of the gene marker is linked with the potential phenotype of MUT. Nonetheless, gene marker-based phylogenies are not always congruent with organismal phylogenies (i.e., due to horizontal gene exchange between distantly related bacteria or evolution of functionally slightly different enzymes in the same organism; Friedrich, 2002). In general, *mxaF*-based phylogenies correlate with organismal phylogenies on the level of families of methylotrophs (Kist and Tate, 2013a, b; Lau et al., 2013). However, for other genes (*mdh2*, *mdo*, *mdh*, *mod1*, *mod2*, *mtaC*) of methanol-oxidizing enzymes, congruence with organismal phylogenies needs to be evaluated.

**Table 4:** Use of amplicon pyrosequencing to analyze methylotrophic communities.

Environment	Gene markers	Remarks	Functional group	Reference
**Soils**
Aerated soils	*mxaF*, *mch*, *fae1*	Amplification with adapter-less primers	Bacterial methylotrophs	Stacheter et al. (2013)
Hydromorphic grassland soil	*pmoA*	–	Methanotrophs	Shrestha et al. (2012)
Peatland	*pmoA*		Methanotrophs	Deng et al. (2013)
Paddy soils	*pmoA*	–	Methanotrophs	Lüke and Frenzel (2011)
Peat bog	*pmoA*	Read length > 500 nt, blended analysis with other genes	Methanotrophs	Kip et al. (2011)
**Aquatic habitats**
Lake sediments and waters	16S rRNA genes	Combined with DNA SIP	Methanotrophs	He et al. (2012)
Water of oil sand tailings ponds	*pmoA*	–	Methanotrophs	Saidi-Mehrabad et al. (2013)
**Aquifer**
Aquifer	16S rRNA genes	V4–V6 region of 16S rRNA	Methylotrophs (and others)	Lavalleur and Colwell (2013)
**Technical systems**
Methanotrophic biofilter	16S rRNA genes	–	Methanotrophs (and others)	Kim et al. (2013)

Recent evaluation of phylogenetic resolution of *mxaF* compared to organismal phylogenies revealed contradicting results (Kist and Tate, 2013a, b; Lau et al., 2013). The congruency with the 16S rRNA gene phylogeny and the resolution of *mxaF* is sufficient (except for some “anomalies”) in the non-methanotrophic genus *Methylobacterium* (affiliates with Alphaproteobacteria), i.e., the so-called pink-pigmented, facultatively methylotrophic (PPFM) bacteria (Kist and Tate, 2013a, b), and *mxaF*-based taxonomic resolution might be even higher than that of 16S rRNA genes (Kist and Tate, 2013b). Nonetheless, strain-level identification is not possible and requires the analysis of more variable genomic regions (Knief et al., 2008, 2010b). Some alphaproteobacterial genera harbor *mxaF*-like genes that are similar to those of *Methylobacterium* suggesting the occurrence horizontal gene transfer events in evolution of methylotrophs; *Methylobacterium nodulans* ORS 2060A carries a plasmid with methylotrophy genes including *mxaF*, suggesting that this species has acquired this gene from another PPFM bacterium (Kist and Tate, 2013a). Using *mxaF* as a phylogenetic marker of methanotrophic Proteobacteria revealed that the three major families Methylococcaceae, Methylocystaceae, and Beijerinckiaceae can be unambiguously reconstructed (Lau et al., 2013). Nonetheless, *mxaF* and 16S rRNA gene phylogenies differ on genus and species level (Lau et al., 2013). The loose coupling of *mxaF *phylogenies with 16S rRNA gene phylogenies is reflected by a low DNA-level similarity (about 77%) that relates to a 97% similarity cut-off on 16S rRNA gene sequence level, which is indicative for species. This low *mxaF* cut-off level even decreases when more species are considered (Stacheter et al., 2013) supporting the conclusion that horizontal gene transfer occurred between methylotrophs and non-methylotrophs. A frequently detected *mxaF* genotype in temperate aerated soils is closely related to the methanotroph *Methyloferula stellata* AR4. Nonetheless, due to lack of congruencies between *mxaF* and 16S rRNA gene phylogenies in Beijerinckiaceae, it cannot be judged if this *mxaF* genotype was derived from a methanotroph or a non-methanotrophic methylotroph (Lau et al., 2013; Stacheter et al., 2013). Thus, a more in-depth analysis of bacteria that harbor MxaFI-, XoxF-, and Mdh2-like MDHs on the level of genomes is warranted to improve the understanding of the role of horizontal gene transfer and convergences in these organisms aiming at a more correct interpretation of *mxaF*, *xoxF*-like, and mdh2 datasets retrieved from amplicon-based NGS.

## FUTURE PERSPECTIVES

In the era of metagenomics, -transcriptomics, and -proteomics, it is noteworthy to state that the knowledge on diversities of methanol-oxidizing enzymes will also facilitate the detection of such organisms and their metabolic pathways in “omic” datasets. An example is the detection of an actinobacterial MDO-like protein in a metaproteome of rice plants (Knief et al., 2012a). Since the Mdh2 or fungal MDOs have a broad substrate spectrum and may utilize alternative substrates (Nakagawa et al., 2006; Kalyuzhnaya et al., 2008; Gvozdev et al., 2012), their role in *in situ* methanol-oxidation based solely on the detection of their genes is ambiguous when their activity dependent on methanol *in situ* cannot be demonstrated. Moreover, many methylotrophs are capable of utilization of multicarbon compounds. Thus, the detection of a genotype does not necessarily mean that the respective microorganism was involved in methanol oxidation *in situ*. Hence, approaches combining gene marker-based nucleic acid stable isotope probing (SIP; Antony et al., 2010) or even together with protein SIP (Jehmlich et al., 2010) are promising to detect active methanol utilizers in terrestrial and other environments, and may allow for detection of novel oxidoreductases and microorganisms that utilize methanol in the environment. Still not resolved issues for a comprehensive detection of methanol utilizers are (a) the unresolved quantitative impact of organisms that employ other methanol-oxidizing enzymes than methanol-specific oxidoreductases, and (b) the detection of methanol utilizers that dissimilate but do not assimilate methanol-derived carbon. Such organisms might be detectable by SIP when using their actual carbon substrate as a source of isotope label combined with unlabeled methanol and a control, in which the labeled substrate is not supplemented.

## Conflict of Interest Statement

The authors declare that the research was conducted in the absence of any commercial or financial relationships that could be construed as a potential conflict of interest.
